# A new planar feeding method of dielectric rod antenna using dielectric resonator

**DOI:** 10.1038/s41598-023-36543-0

**Published:** 2023-06-07

**Authors:** Saeed Fakhte

**Affiliations:** School of Electrical and Computer Engineering, Qom University of Technology, Qom, Iran

**Keywords:** Engineering, Electrical and electronic engineering

## Abstract

This article proposes a new method for exciting surface waves in dielectric rod antennas using dielectric resonator antennas. The method involves housing a rectangular dielectric resonator antenna with a dielectric constant of 10.2 inside a hollow cylindrical dielectric rod antenna made of Teflon. By exciting the ${TE}_{111}^{y}$ and ${TE}_{113}^{y}$ modes of the dielectric resonator antenna, a surface wave can be launched along the Teflon tube. This method offers the advantage of integrating the dielectric rod antenna with planar circuits, where maximum radiation in the direction normal to the board is desirable. Compared to other planar feeding methods, this technique leads to lower back lobe and sidelobe levels. I fabricated the proposed structure and conducted tests to measure its performance. The measured results show an impedance bandwidth of 22% from 7.35 to 9.4 GHz and a maximum gain of 14 dB. Additionally, the simulated radiation efficiency of the proposed antenna in the entire band is above 90%.

## Introduction

Dielectric rod antennas are excellent candidates for use in the millimeter wave band due to their wide impedance bandwidth, high gain, stable radiation pattern, and high radiation efficiency^1–3^. The minimal use of metal in the antenna structure contributes to its high radiation efficiency. At low frequencies, these antennas are used as feed systems for reflector antennas^4^, while in the millimeter wave range, they are directly integrated with circuits. Numerous theoretical and experimental studies have been conducted to understand the working mechanism and predict the radiation properties of these antennas^5,6^. Due to their many positive features in both microwave and millimeter wave frequency bands, they have become widely used in various telecommunication applications^7–10^. Recently, they have been introduced as efficient antennas in terahertz and optical bands for on-chip interconnection communications between integrated circuit elements^11–14^. However, integrating them with planar circuits is a challenge since a non-planar waveguide structure is required to excite this antenna.

Dielectric rod antennas can be excited in various ways, with both planar and non-planar structures. For instance, in^15^, a V-shaped twin-wire tapered transverse electromagnetic waveguide was utilized to feed the dielectric rod antenna. Although this feeding structure has created a wide impedance bandwidth, it lacks the ability to integrate into planar structures. In another study^16^, a square metal waveguide and a conical horn were used to excite the dielectric rod antenna. Additionally, two perpendicular microstrip-to-waveguide transitions were employed to excite the waveguide, resulting in the excitation of two orthogonal modes in the waveguide. Several other works can be found in the literature where dielectric rod excitation was done with metallic waveguides or other non-planar feeding networks; however, they are not reviewed here for brevity^17–23^.

In certain applications, such as mm-wave base stations and automotive radar, a planar feeding structure is necessary to excite the antenna. This method allows for easier arraying of the dielectric rod antenna. For instance, in^24^, the authors fed the antenna using a folded slot aperture in the ground plane. However, this method has limited application due to the back lobe in the radiation pattern. In this article, we will compare this feeding method with our proposed method. Other similar works have also used slot excitation^25^. There are alternative planar feeding methods that result in an end-fire radiation pattern^26–28^.

In our work, we introduce a novel approach where a dielectric resonator antenna (DRA) is used to feed the dielectric rod antenna for the first time. A rectangular DRA is placed inside a long Teflon tube and excited from below by a slot aperture. The use of DRA enhances electromagnetic coupling from microstrip line to the dielectric rod while minimizing back radiation of the antenna. The key difference between this method and feeding by slot aperture lies in our ability to optimize side lobes and back lobe levels of the radiation pattern.

 The paper is organized as follows: in “Antenna configuration” the antenna configuration is presented. In “Parametric study” a parametric study is conducted. In “Comparison with slot fed dielectric rod” the proposed feeding method is compared with the previously reported slot excitation method. In “Measurement results” the measured and simulated results of the proposed antenna are compared with each other. Finally, in “Conclusion” the conclusion of the paper is presented.

## Antenna configuration

**Figure 1:**
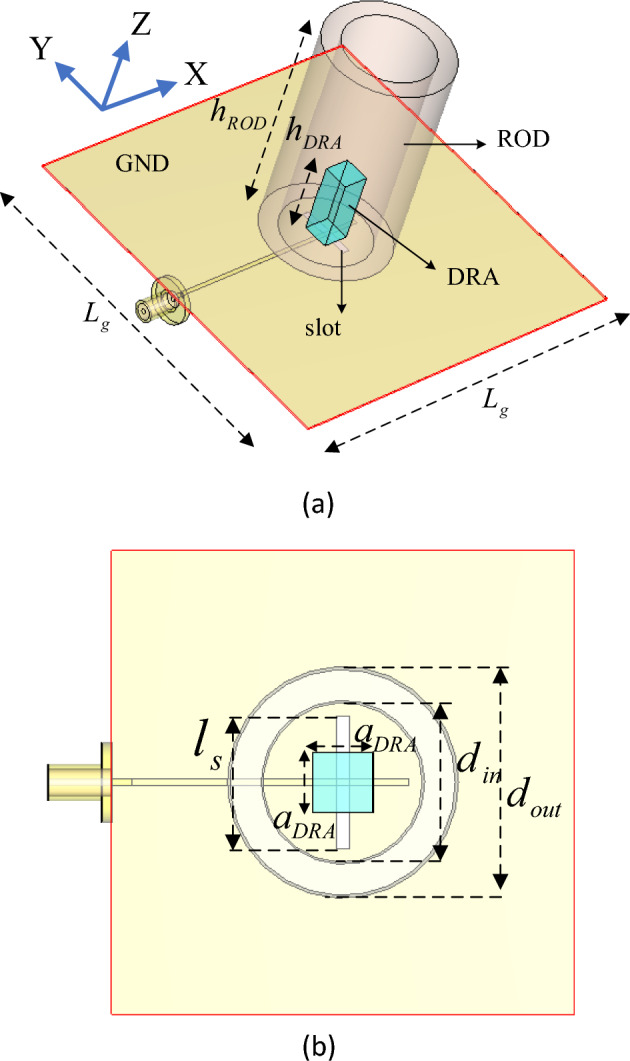
Geometry of the proposed antenna, (**a**) 3D view, (**b**) top view.

Figure 1 shows the geometry of the proposed dielectric resonator fed dielectric rod antenna. As can be seen, a rectangular DRA with dimensions of $a_{DRA} \times a_{DRA} \times h_{DRA}$ is housed inside a long teflon tube that plays the role of a dielectric rod. The inner and outer diameters and the height of teflon tube are $d_{in}$, $d_{out}$, and $h_{ROD}$, respectively. A slot aperture with dimensions $l_{s} \times w_{s}$ is engraved in ground plane with the size of $L_{g} \times L_{g}$. The slot aperture is excited from below by a microstrip feed line and used to couple the power from the line to the DRA. The microstrip line is printed on the RO 4003 substrate with a thickness of 0.508 mm and a dielectric constant of 3.55. The DRA is fabricated using Rogers 6010 dielectric material, which has a dielectric constant of 10.2 and a loss tangent of 0.0023.

The operational frequency band of the antenna is determined by the DRA and slot modes. To determine the DRA resonance frequency, it can be assumed that the Teflon tube does not have a significant effect on the DRA resonance frequency due to its distance from the DRA. Therefore, to calculate the resonance frequency of DRA, the well-known dielectric waveguide method^29,30^ can be used to calculate the resonance frequency of rectangular DRA. To excite $TE_{111}^{y}$ mode in DRA at 8 GHz frequency, DRA dimensions are obtained as aDRA = 7.1 mm and hDRA = 19.3 mm. All antenna parameters are optimized to achieve the best gain, sidelobe level, bandwidth and back lobe level. The optimized values of these parameters are listed in Table 1. The simulations are done using CST MW Studio 2022.

**Table 1:** The optimized values for the parameters of proposed antenna.

Parameter	$a_{DRA}$	$h_{DRA}$	$d_{in}$	$d_{out}$	$l_{s}$	$h_{ROD}$
Value (mm)	7.1	19.3	16	28	5.16	74

**Figure 2:**
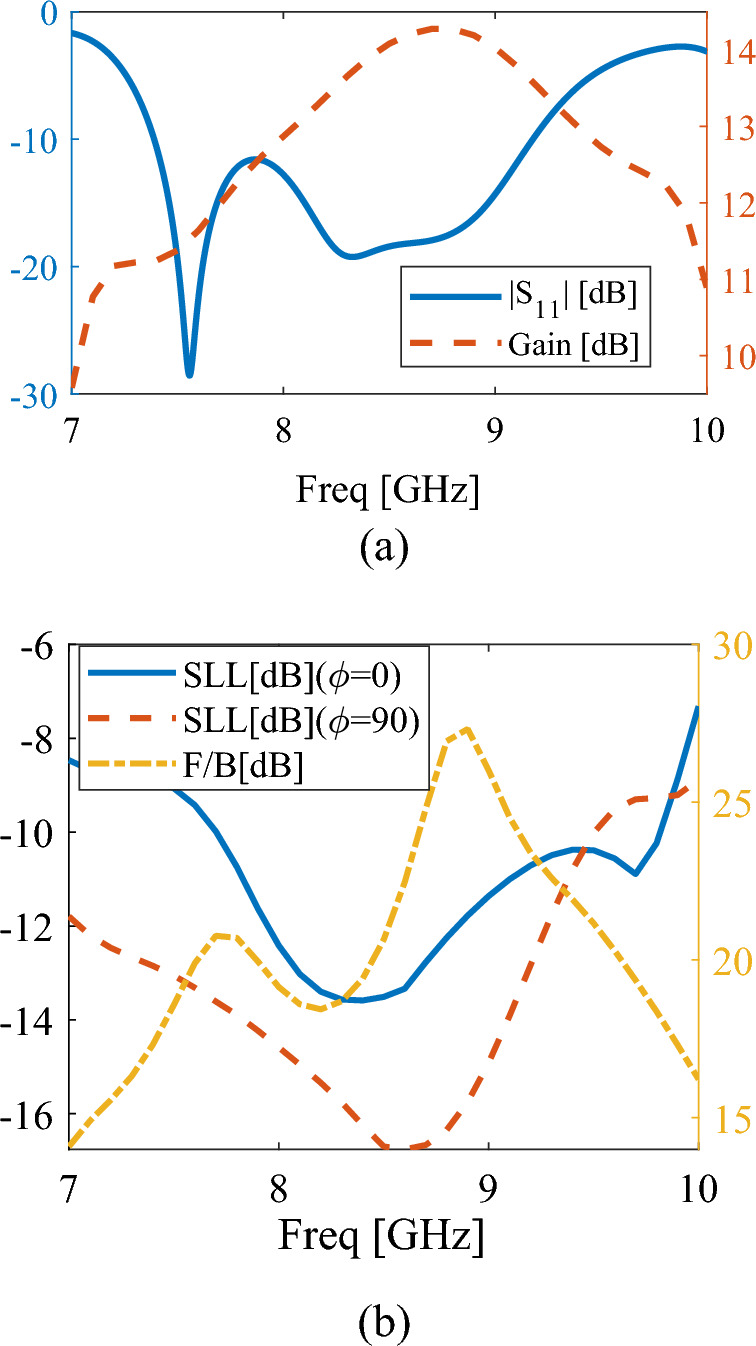
The simulated results of proposed antenna, (**a**) reflection coefficient and gain of proposed antenna, (**b**) side lobe levels in XoZ and YoZ planes and front-to-back ratio.

Figure 2a shows the simulated reflection coefficient of the proposed antenna. Three resonances can be seen in the reflection coefficient curve, which result from the resonance of DRA and slot modes. Also, from the antenna gain diagram shown in this figure, it can be seen that a gain above 13 dB has been obtained in the frequency range that is related to the resonance of DRA modes. Figure 2b shows the SLL and front to back ratio (F/B) diagrams, which indicate a good performance for this antenna in the frequency range of 7.5 to 9.5 GHz. In particular, it can be seen that the F/B ratio is very good, which indicates a significant reduction in the back lobe level of radiation pattern.

Before the parametric study of the antenna, let's plot the electric field distributions at three frequency points associated with the minima of the reflection coefficient diagram to determine which frequencies are associated with the resonance of the DRA and slot modes.

**Figure 3:**
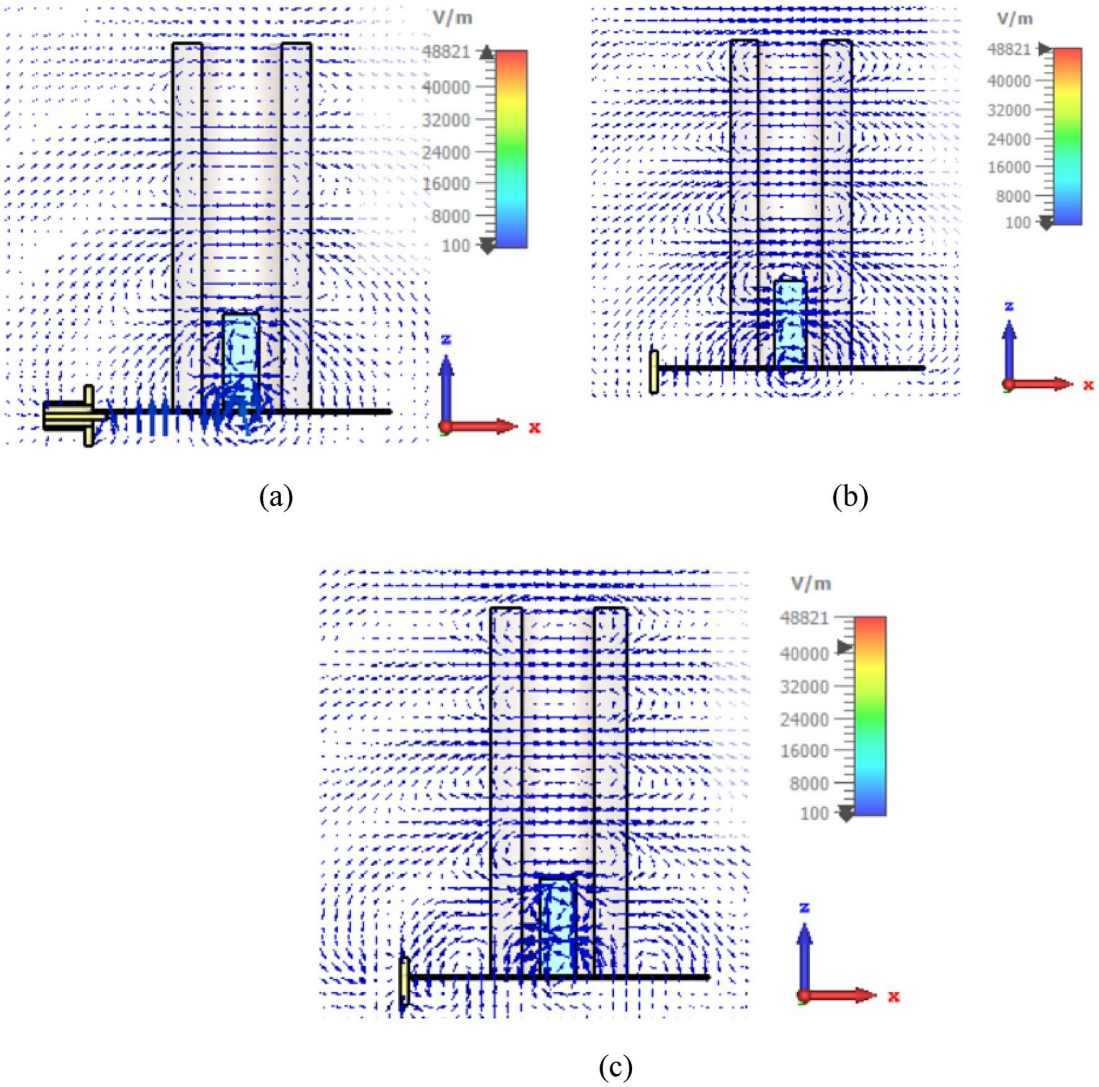
The simulated electric field distributions of proposed antenna, (**a**) at 7.6 GHz, (**b**) at 8.3 GHz, and (**c**) at 8.8 GHz.

As shown in Fig. 3a, at the frequency corresponding to the first minimum in the reflection coefficient diagram, i.e. 7.1 GHz, the magnitude of the electric field around the slot is strong, which indicates the resonance of the mode is related to the slot mode. Also, in the second and third minimum frequencies of the diagram, i.e. 8.3 and 8.8 GHz, the electric field distributions shown in Fig. 3b, c resemble the $TE_{111}^{y}$ and $TE_{113}^{y}$ modes of DRA.

Now that the origin of the resonances is known, another thing that can be seen in these figures is the excitation of surface waves inside the Teflon tube. By comparing Fig. 3b,c with Fig. 3a, it can be understood that the excited waves in the tube are stronger when the sources of resonance are DRA modes. DRA seems to cause the wave to be pulled up.

## Parametric study

**Figure 4:**
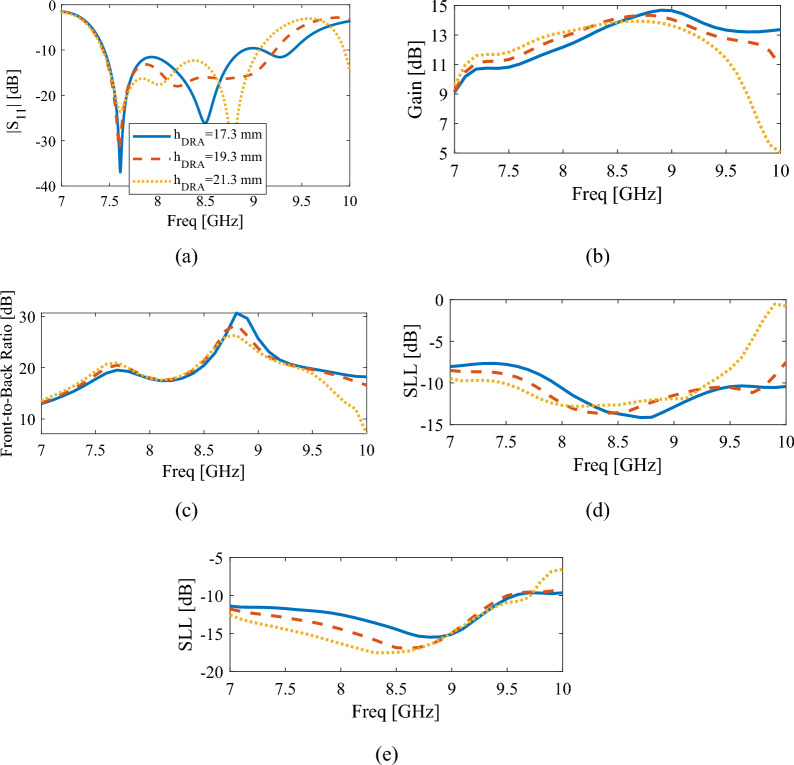
The simulated results of the proposed antenna for different values of DRA height, $h_{DRA}$, (**a**) reflection coefficient, (**b**) gain, (**c**) front-to-back ratio, (**d**) side lobe level in XoZ plane, (**e**) side lobe level in YoZ plane.

This section includes parametric studies of the antenna behavior, with variations on the dielectric resonator height and width, dielectric rod height and diameters. First, the influence of DR height on antenna characteristics has been investigated. It can be seen in Fig. 4a that with the change of DR height, the location of the two upper minima in the reflection coefficient curve changes, while the location of the first minimum does not change significantly, which indicates that the first resonance is not related to the DRA mode, but to Slot mode.

**Figure 5:**
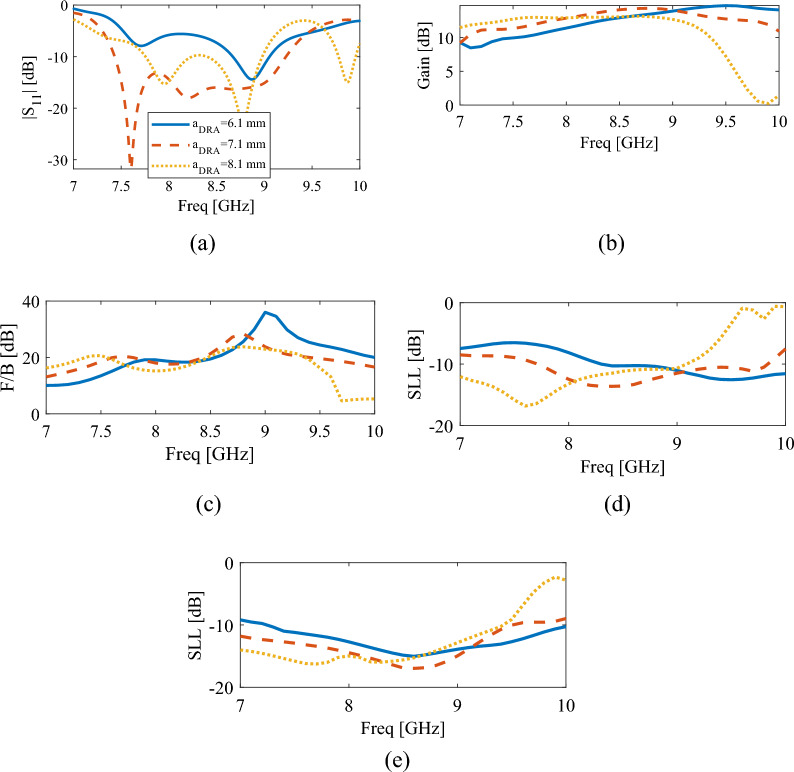
The simulated results of the proposed antenna for different values of DRA length, $a_{DRA}$, (**a**) reflection coefficient, (**b**) gain, (**c**) front-to-back ratio, (**d**) side lobe level in XoZ plane, (**e**) side lobe level in YoZ plane.

Figure 4b–e show the changes of gain, front-to-back ratio, and sidelobe levels for *h*_*DRA*_ between 17.3 and 21.3 mm. Observe that the best possible choice is h = 19.3 mm. Also, it is found out that the best SLL in both XoZ and YoZ planes, gain and front-to-back ratio, is obtained for the frequency range that the DRA resonates, that is, in the upper part of the operational band. In fact, as mentioned earlier, this is the advantage of using DR to excite the dielectric rod instead of a single slot. Figure 5 shows the influence of DR length on the output results of the proposed antenna. Changing the length of DR changes the location of the two upper minima of the curve. Increasing the length of DR causes the frequency of these two points to decrease, which happens due to the change in the resonance frequency of the TE111 and TE113 modes of the DR antenna. Also, the location of the first minimum of the curve, which is related to the resonance of the slot, does not change, and only its level has changed. From Fig. 5b–e which show the graphs of gain, front to back ratio, and sidelobe levels in XoZ and YoZ planes, it can be understood that to make a compromise between these results, the DR length should be 7.1 mm.

**Figure 6:**
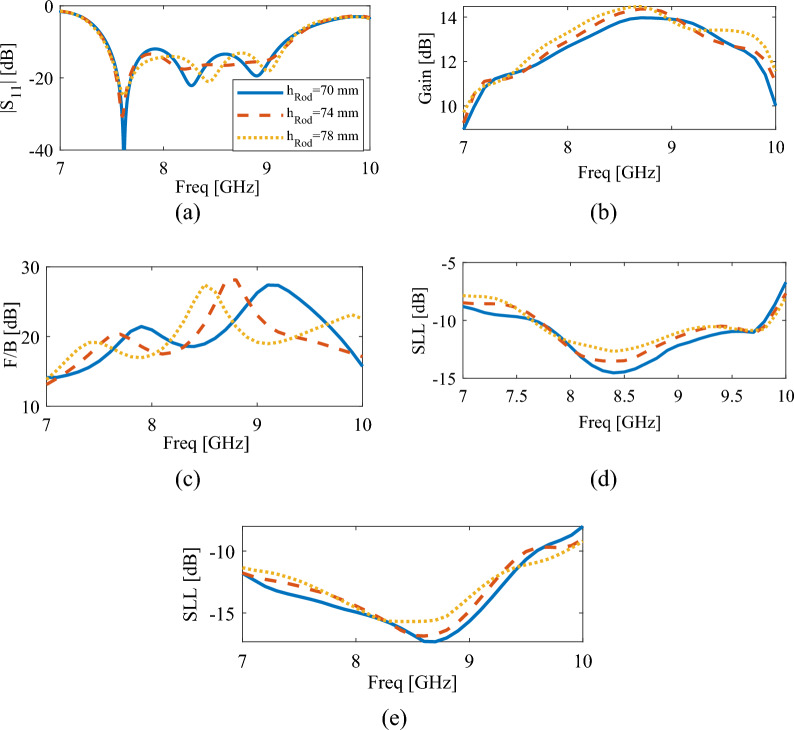
The simulated results of the proposed antenna for different values of dielectric rod height, $h_{Rod}$, (**a**) reflection coefficient, (**b**) gain, (**c**) front-to-back ratio, (**d**) side lobe level in XoZ plane, (**e**) side lobe level in YoZ plane.

The changes of reflection coefficient, gain, front-to-back ratio, and sidelobe levels of the antenna with variation in rod height are shown in Fig. 6. It can be seen from Fig. 6a that the bandwidth of the antenna has not changed significantly. This is because the three resonances in the operational band originate from the DR and slot modes. But from Fig. 6b–e it is clear that the height of the rod has a significant effect on the far field results of the antenna and a good compromise between these outputs is reached for *h*_*Rod*_ = 74 mm. As shown in Fig. 6c, changing the height of the rod has a significant effect on the frequency locations of the front-to-back ratio (F/B) peaks. However, the locations of |S11| remain unchanged. This indicates that the presence of the rod causes the frequency locations of the F/B peaks to not coincide with |S11|. The F/B curve in this figure also shows that as the rod height increases to 78 mm, three peaks appear in the F/B curve at frequencies of 7.5, 8.5 and 9.8 GHz. These peaks correspond to the resonance of the slot and the two resonances of the DRA modes.

## Comparison with slot fed dielectric rod

**Figure 7:**
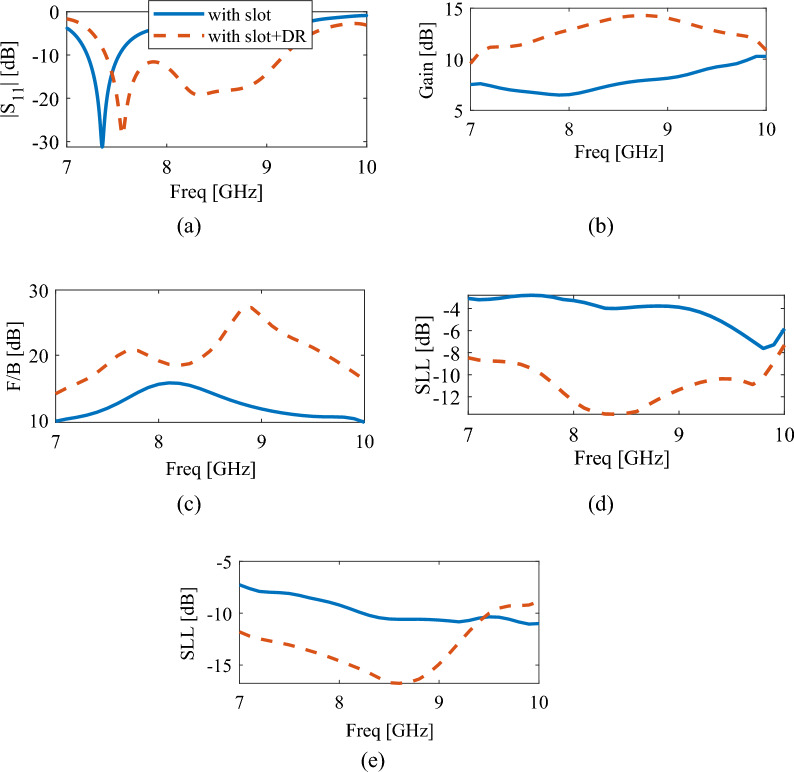
Comparison between simulation results of dielectric rod antenna coupled to DR with rod antenna coupled to slot aperture, (**a**) reflection coefficient, (**b**) gain, (**c**) front-to-back ratio, (**d**) side lobe level in XoZ plane, (**e**) side lobe level in YoZ plane.

In this section, the comparison between the simulation results of the dielectric rod antenna coupled to DR and the dielectric rod antenna coupled to the slot aperture is discussed. Figure 7 shows a comparison between reflection coefficients, gains, front-to-back ratios and SLLs of these two antennas. The superiority of the DRA-coupled antenna is evident in all these curves.

**Figure 8:**
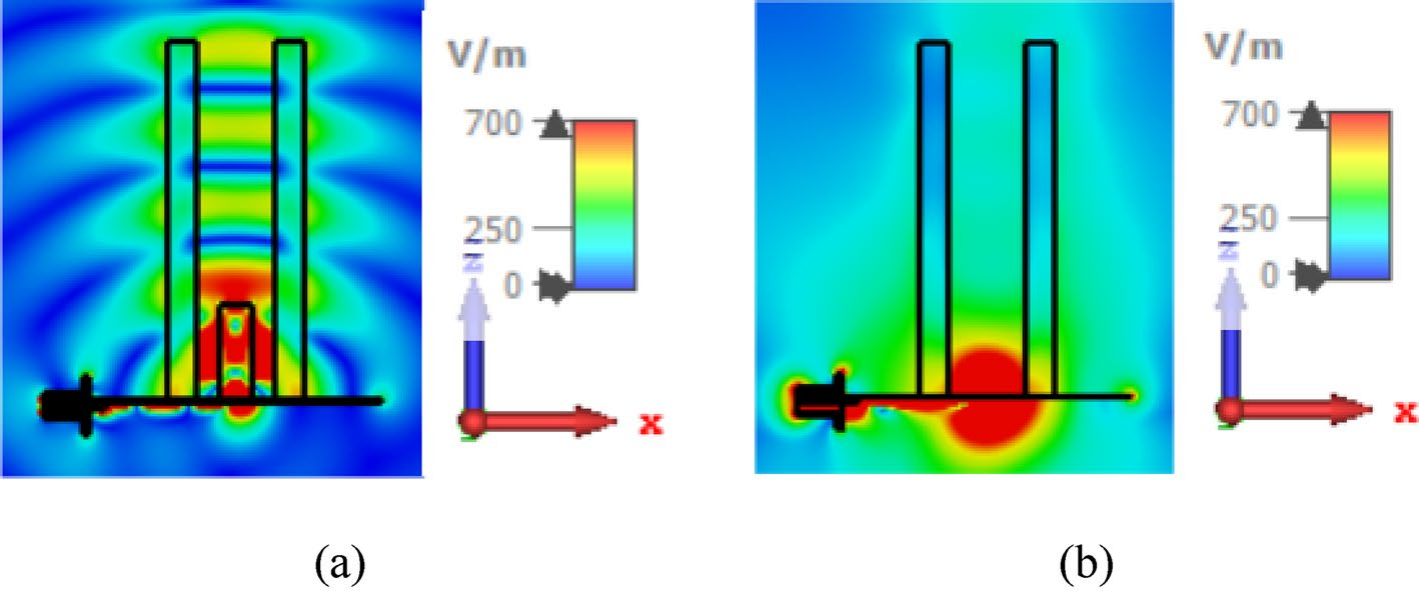
The electric field distribution of dielectric rod antenna with two different feeding methods, (**a**) coupled to DR at 8.4 GHz, (**b**) coupled to slot aperture at 7.35 GHz.

To explain the reason for this superiority, the electric field distributions of the two antennas are shown in Fig. 8, which shows the strong coupling of the electric field to the DRA-fed Teflon tube compared to the slot-fed tube. In fact, the DRA has caused the waves to be directed upwards, compared to the slot, which tends to have relatively equal radiation both upwards and downwards.

The power that is transferred between the port and the antenna is influenced by the port’s type and position relative to the dielectric rod antenna. While numerical methods are typically required to determine these values, understanding the field distributions of the rod’s modes and using the Lorentz Reciprocity Theorem can provide valuable insights. The source can be represented as either an electric or magnetic current when coupling to a rod, and the reciprocity theorem can be used with the appropriate boundary conditions to determine the coupling amount, χ, between the source and the rod’s fields. The amount of coupling for electric and magnetic sources can be calculated with the following equations^31^.

1 $$ \chi \propto \int_{V} {\left( {{\varvec{E}}_{ROD} \cdot {\varvec{J}}_{s} } \right)dV} $$

2 $$ \chi \propto \int\limits_{V} {\left( {{\varvec{H}}_{ROD} \cdot {\varvec{M}}_{s} } \right)dV} $$

where Js and Ms represent electric and magnetic current sources, respectively and EROD and HROD refer to the electric and magnetic fields within the dielectric rod. V represents the volume where the electric and/or magnetic current sources are present.

According to Eq. (1), to achieve strong coupling with an electric current source, the source should be placed in an area with strong electric fields within the rod. Conversely, Eq. (2) states that to achieve strong coupling with a magnetic current source (such as a loop or aperture), the source should be placed in an area with strong magnetic fields. In these two equations, it is evident that the larger the volume of V, representing the volume of sources interacting with the rod fields, the greater the amount of coupling.

**Figure 9:**
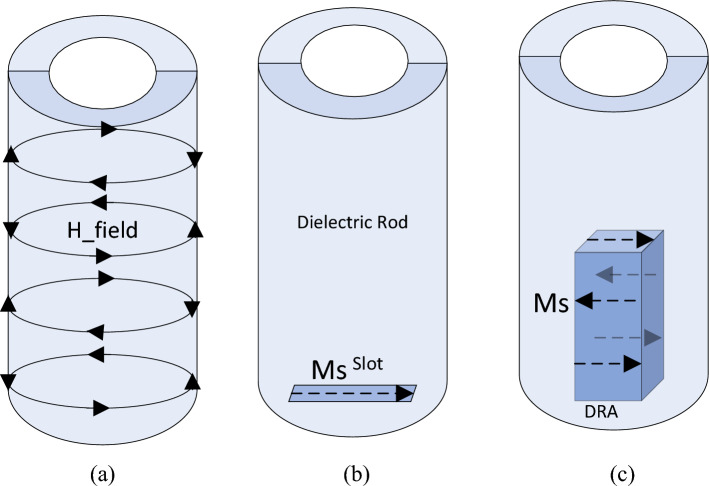
Simple modeling of slot and DRA as sources of magnetic currents inside dielectric rod, (**a**) magnetic fields of travelling waves inside dielectric rod, (**b**) slot aperture as a magnetic current source, (**c**) equivalent magnetic current densities over the walls of DRA.

An aperture slot can be considered as a source of magnetic current. It has also been demonstrated that a DRA can be modeled using magnetic current sources on its walls. While electric currents also exist on the walls when modeling with the equivalence principle, their range is small compared to the equivalent magnetic currents and can be disregarded^32,33^. Figure 9 shows a simplified model of the DRA and slot inside the dielectric rod. It can be observed that the volume in which the DRA’s magnetic equivalent currents interact with the dielectric rod’s magnetic field is larger than the volume of the slot’s magnetic current engagement. As a result, Eqs. (1) and (2) indicate that the amount of wave coupling from the DRA to the rod is greater than that from the slot to the rod.

## Measurement results

**Figure 10:**
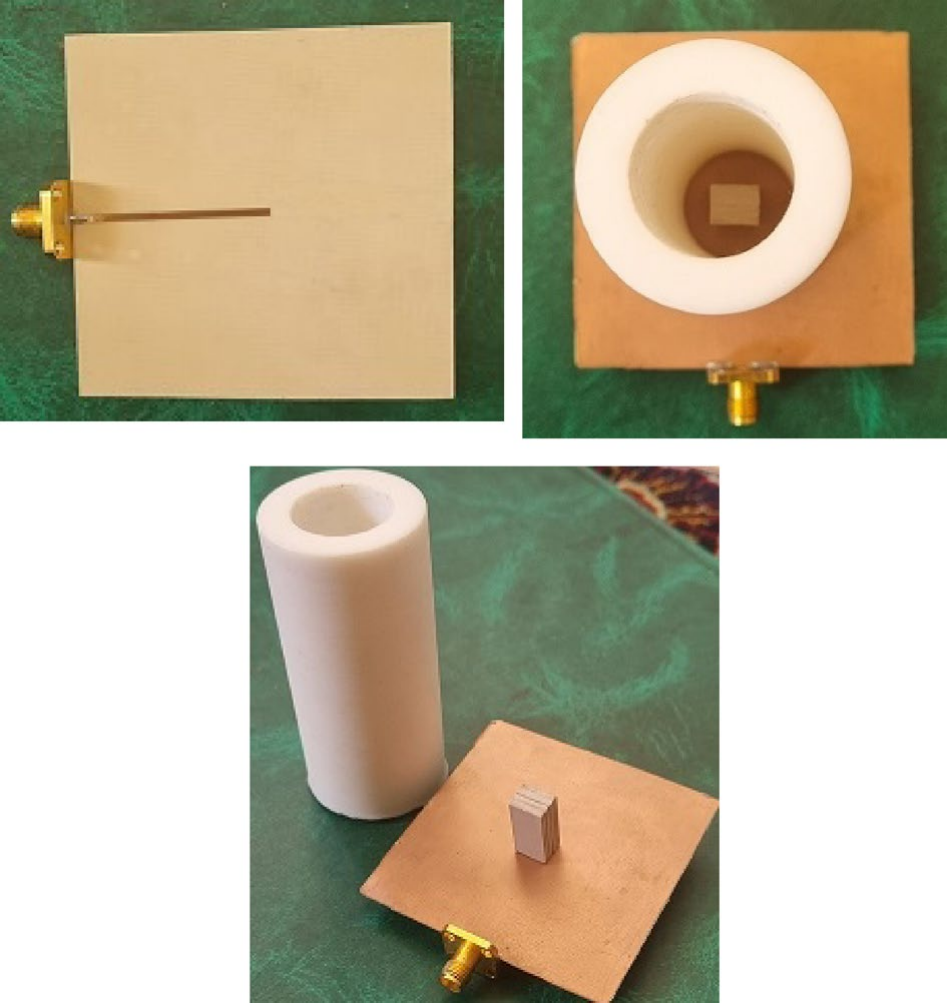
The fabricated prototype of the proposed dielectric rod antenna.

**Figure 11:**
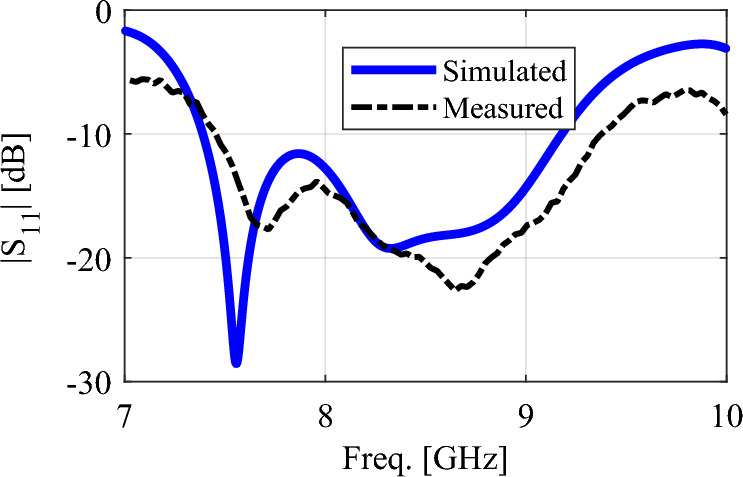
The simulated and measured reflection coefficients of the proposed antenna.

To confirm the simulation results, a prototype of dielectric rod antenna fed with DRA is fabricated, which is shown in Fig. 10. The antenna was tested in the antenna laboratory of KNTU University in Tehran. A HEWLETT-PACKARD 8410C NETWORK ANALYZER was used to measure the antenna’s reflection coefficient. A standard WR-102 horn antenna, which covers the frequency band of 7 to 11 GHz, was used for the test. The results of the reflection coefficient obtained from the simulation and measurement are shown in Fig. 11, which shows an impedance bandwidth of 22% from the frequency of 7.45 to 9.3 GHz. There is some discrepancy between the simulation and measurement results, which is due to manufacturing and measurement errors. The measurement error is caused by the presence of the power cable and other devices around the antenna under test.

**Figure 12:**
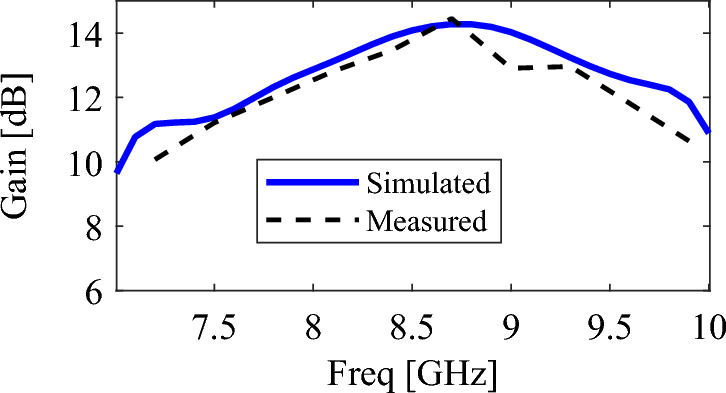
The simulated and measured gains of the proposed antenna.

Figure 12 shows the simulation and measurement gains. As can be seen, the maximum gain is 14 dB and the gain in the whole band is higher than 11 dB. A good agreement between the measurement and simulation results is achieved. It should be noted that by increasing the length of the rod up to $6\lambda_{0}$, the gain can be increased even more than this value, but the purpose of this article is to prove a new feeding method for the rod antenna and a structure with a lower height of about $2\lambda_{0}$ is considered.

**Figure 13:**
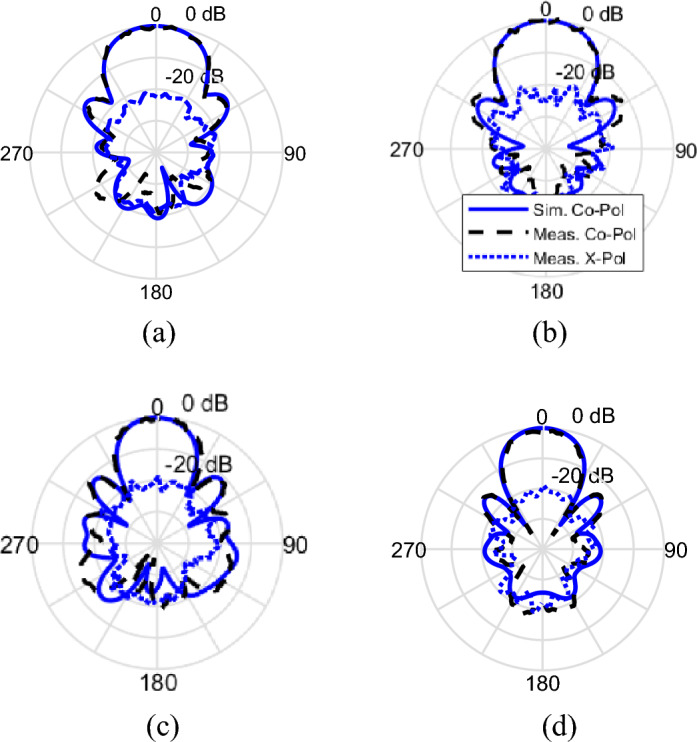
The simulated and measured radiation patterns of the proposed antenna, (**a**) at 8 GHz in XoZ plane, (**b**) at 8 GHz in YoZ plane, (**c**) at 9 GHz in XoZ plane, (**d**) at 9 GHz in YoZ plane.

Figure 13 shows the radiation patterns of the proposed antenna at 8 and 9 GHz in both XoZ and YoZ planes. It can be observed that the radiation patterns of the antenna are directed towards the boresight and the sidelobe levels of the antenna are below 10 dB. The cross polar discrimination of more than 20 dB is attained. Also, the measurement results are in relatively good agreement with the simulation results.

Finally, the proposed antenna was compared to other dielectric rod structures that also exhibit a broadside radiation pattern. It is important to note that the use of a dielectric resonator to feed the dielectric rod was intended to create a structure with a broadside pattern. This means that the main radiation pattern lobe is perpendicular to the ground plane. As a result, it is not appropriate to compare this structure with samples that have an end-fire radiation pattern. This is because, as previously mentioned, having a broadside pattern in the antenna offers many advantages over end-fire patterns, including the ease of arraying the antenna. There are only a few reported examples of dielectric rod antennas with broadside patterns, and there is still room for further research in this area. Another advantage of this antenna is its completely planar feeding network. This eliminates the need for a metal enclosure in the initial section of the dielectric rod, which is present in many previous works.

Table 2 presents a comparison between the proposed antenna and previously reported antennas that exhibit a broadside pattern. Compared to the work presented in^24^, the proposed structure has a relatively equal impedance bandwidth, but its gain is lower. One reason for this is that the structure reported in^24^ is a 2 × 2 array of DRAs. However, as shown in the table, the length of the work presented in^24^ is almost three times that of our work.

**Table 2:** Comparison with other reported dielectric rods with planar feeding method (${\lambda }_{g}={\lambda }_{0}/\sqrt{{\varepsilon }_{r}}$ in the middle of the frequency band).

Ref	Structure	Feed type	Pattern type	Antenna length (${\lambda }_{g}$)	Impedance bandwidth (%)	Max gain (dB)
^24^	2*2 array	Folded slot	Broadside	8.7	28	19
^25^	Slot fed DRA	Slot	Broadside	5.08	40	11.5
^34^	Patch with waveguide	Patch + waveguide	Broadside	8.7	5	16
^35^	Slot with waveguide	Slot + waveguide	Broadside	> 2.4	11	12
Proposed work	DRA fed rod	DRA + slot	Broadside	3	22	14

Compared to the work presented in^25^, our proposed work has a smaller electrical dimension and a higher gain. The impedance bandwidth of the proposed work is smaller than that of^25^. However, it is important to note that the bandwidth in the proposed structure can be increased by using a broadband dielectric resonator.

In works^34^ and^35^, planar slot and patch feeds are used, but they are surrounded by a waveguide, which increases the complexity of construction. However, as shown in the table, work^34^ has a very small impedance bandwidth, and work^35^ also has a lower impedance bandwidth and gain than the proposed work.

## Conclusion

This article presents a planar structure for exciting surface waves in a dielectric rod antenna. The structure allows for control of the side lobe and back lobe levels in the antenna radiation pattern. This method is useful for feeding the rod antenna when a directional pattern perpendicular to the ground is needed. Compared to using a slot to excite the rod, using a dielectric resonator reduces back lobe and side lobe levels in the radiation pattern, while also achieving higher impedance bandwidth and gain. The proposed antenna has been fabricated and measurement results confirm simulation results. The antenna has an impedance bandwidth of 22% and a maximum gain of 14 dB as measured.

## Data Availability

The datasets used and/or analyzed during the current study available from the corresponding author on reasonable request.
